# Activin A and Sertoli Cells: Key to Fetal Testis Steroidogenesis

**DOI:** 10.3389/fendo.2022.898876

**Published:** 2022-05-24

**Authors:** Liza O’Donnell, Penny A. F. Whiley, Kate L. Loveland

**Affiliations:** ^1^ Centre for Reproductive Health, Hudson Institute of Medical Research, Clayton, VIC, Australia; ^2^ Monash University, Clayton, VIC, Australia

**Keywords:** androgens, activin A, Sertoli cell, Leydig cell, fetal steroidogenesis

## Abstract

The long-standing knowledge that Sertoli cells determine fetal testosterone production levels is not widespread, despite being first reported over a decade ago in studies of mice. Hence any ongoing use of testosterone as a marker of Leydig cell function in fetal testes is inappropriate. By interrogating new scRNAseq data from human fetal testes, we demonstrate this situation is also likely to be true in humans. This has implications for understanding how disruptions to either or both Leydig and Sertoli cells during the *in utero* masculinization programming window may contribute to the increasing incidence of hypospadias, cryptorchidism, testicular germ cell tumours and adult infertility. We recently discovered that activin A levels directly govern androgen production in mouse Sertoli cells, because the enzymes that drive the conversion of the precursor androgen androstenedione to generate testosterone are produced exclusively in Sertoli cells in response to activin A. This minireview addresses the implications of this growing understanding of how *in utero* exposures affect fetal masculinization for future research on reproductive health, including during programming windows that may ultimately be relevant for organ development in males and females.

## Dogma Relating to Fetal Testis Biology: Moving and Stationary Targets

More than 30 years ago, the events that lead to gonad masculinization were shown to be triggered by the expression of *SRY* in pre-Sertoli cells of the fetal testis, in studies of humans (1) and mice (2). Since that time, we have learned just how complicated that process is, including the contribution of signals relating to SOX9 feedback (3), growth factors (4, 5) and retinoic acid (6). These cues direct primordial germ cells to differentiate into the earliest sperm precursors and instruct the somatic cells to multiply, differentiate, and form the testis cords that underpin adult spermatogenesis (7). Our established understanding was that many gonadal cell types were initially bipotential, but subsequently became set in their fate in fetal life. One type became either Sertoli cells in males or granulosa cells in females, providing direct support and instructions to germ cells. However, complementary break-through studies identified that somatic cell fate was actually pliable in both mice and humans. For example, in several studies published over a decade ago, the transcription factor Dmrt1 (Doublesex And Mab-3 Related Transcription Factor 1) was shown to be essential to stabilize the masculinized phenotype of Sertoli and Leydig cells (8), while Foxl2 serves a similar role in sustaining the ovarian phenotype in granulosa and thecal cell lineages (9). The reality of sex-reversal in somatic cell fate was thereby revealed.

Understanding how somatic cells instruct male or female differentiation has also been important for learning about human conditions which can be linked with sub- or infertility, due to impaired germline differentiation or reduced germ cell survival. The conceptualization of ‘Testicular Dysgenesis’ as a spectrum of phenotypes, including hypospadias, cryptorchidism, testicular germ cell tumours and infertility, has provided a basis for mechanistic studies of each of these conditions, as each could result from impaired somatic-germline communication in the fetal testis (10). The Testicular Dysgenesis Hypothesis has stood the test of time; the continuously increasing rates of these conditions worldwide highlights the vulnerability of the developing male reproductive tract to lifestyle and environmental exposures (11), in concert with a lesser, but relevant genetic determinant of disease risk. Mechanistically, this hypothesis has focussed on explaining the impact of disruptions to cell-cell communication in the testis *in utero*, particularly with regard to androgen production and signalling (12).

**Figure 1:**
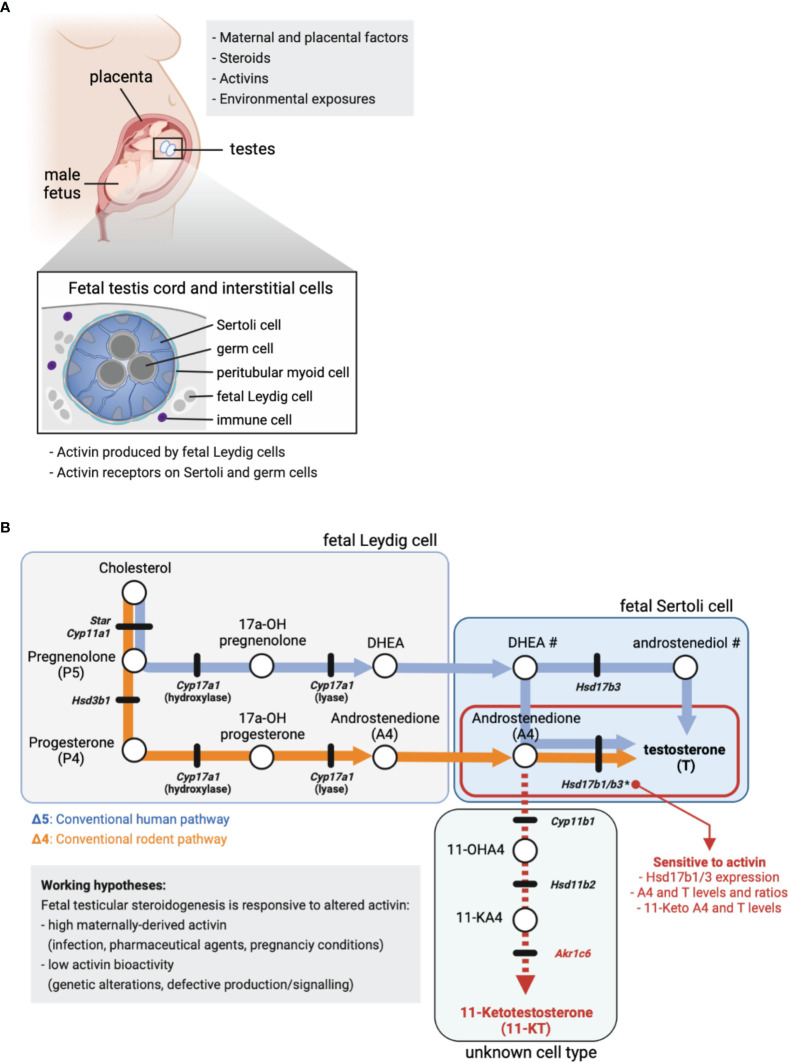
Fetal steroid production. **(A)** Maternal and placental factors, exogenous steroids, activins and environmental exposures each have the potential to alter fetal steroid production. Schematic of placenta, male fetus and testes *in utero*. Cross-section identifies key fetal testis cell types in the cord (germ and Sertoli cells) and in the surrounding interstitium (fetal Leydig, peritubular and immune cells). Activin is produced by fetal Leydig cells and signals *via* specific cell surface receptors present on Sertoli and germ cells. **(B)** Simplified summary of the conventional steroidogenic pathway in human (Δ5, blue line/arrows) and rodent (Δ4, orange line/arrows) fetal testes. The 11-Keto androgen pathway is indicated (red dashed line), as are 11-Hydroxyandrostenedione (11-OHA4), 11-Keto androstenedione (11-KA4) and 11-Ketotestosterone (11-KT). The cellular site of steroid production is indicated, where known. Circles denote cholesterol and steroids, while black lines indicate genes encoding enzymes involved in the conversion of one steroid to the next. Mouse nomenclature has been used throughout; **Table 1** lists human nomenclature. # indicates the local site(s) of DHEA and androstenediol production in human is unclear. * indicates activin A-responsive genes; the ratio of A4 to T in mouse fetal Sertoli cells is dose-dependently reduced by low levels of *Hsd17b1* and *Hsd17b3* expression driven by altered activin A bioactivity. In these testes, excess A4 is associated with higher levels of 11-keto androgens.

Crucial to understanding the origins of testicular dysgenesis outcomes has been delineation of a masculinization programming window (termed MPW), the period of development when the male fetus is sensitive to exposures that impact on reproductive health. During this window, multiple factors can influence the development of the male gonad and secondary sexual characteristics (**Figure 1**). Virilization of the fetal male is dependent on gonadal function but it is also vulnerable to alteration by maternal- and/or placental-derived factors (**Figure 1**). Best understood in rodent models, endocrine disruptor chemical exposures in the interval following sex determination and prior to birth have repeatedly been shown to increase the incidence of conditions including reduced anogenital distance, hypospadias, cryptorchidism and reduced germ cell numbers (13–15). These outcomes have been explored in data collected from boys and men, and from cultures of human fetal testes (16–18), with a growing understanding that not only exposure to endocrine disruptor chemicals, which can also include common pharmaceuticals, may negatively impact fetal and postnatal development. These agents can alter steroid composition and levels and may thereby influence local events in the testis. For example, ibuprofen alters human fetal Leydig cell function and testosterone production between weeks 7–17 of gestation, indicating that human male fetal steroidogenesis is vulnerable to alterations by a commonly used pharmacological compound (16). Nodal and activin signalling can also influence particular aspects of human fetal steroidogenesis in both the first and second trimesters (19), and as discussed below, these members of the TGFβ signalling pathway are amongst the key candidates for driving lifelong changes in male reproductive health.

Activin A is a member of the TGFβ signalling superfamily that is emerging as an important regulator of male fetal development. Activin A was originally identified as a factor that was produced and acted locally to control release of follicle stimulating hormone from the pituitary gland (20, 21). It is synthesized as a disulfide-linked homodimer of two inhibin beta A subunits, encoded by the *Inhba* gene, and its structure and signalling pathway are characteristic of the more than 30 other members of the TGFβ superfamily of signalling molecules, many of which are simultaneously present in the fetal testis (5). It is now known to be produced throughout the body and serve functions in growth and development of many organs, while activin A overproduction is associated with severe pathologies such as cancer cachexia (22). Physiological regulation of activin signalling is complex, occurring through extracellular, transmembrane and intracellular means which are typically context-dependent. See (21, 23–27) for recent reviews on this topic.

Activin A performs multiple functions in testis development that include governance of Sertoli cell proliferation. This role was first revealed in the adult inhibin α knockout mouse strain with elevated activin A; males succumb to Sertoli cell tumours due to the absence of the potent activin inhibitor, inhibin, which forms as a heterodimer of one inhibin α subunit with an inhibin β A subunit (28). In addition to driving Sertoli cell proliferation *in utero*, activin A production by fetal Leydig cells also promotes the timely entry of fetal germ cells into a quiescent state following sex determination (29–31). Quiescence occurs in an important developmental interval, coincident with important aspects of normal male germ cell differentiation that include epigenetic reprograming and loss of pluripotency, each of which can be linked with reducing the risk of tumour formation (32). We have recently discovered that activin A is an important factor regulating androgen biosynthesis in the fetal testis (33); the mechanisms and significance of these findings will be discussed below.

## Learning About Androgen Biosynthesis in the Fetal Testis

### A Gold Mine of Data From Single Cell Transcriptome Analyses

The application of single cell RNA sequencing to study the mammalian fetal testis has revealed developmental relationships between somatic cells which highlight their common origins. Work in mouse (34) and human (35) have detailed the emergence of distinct Leydig and Sertoli cell populations from a single progenitor, extending findings from earlier lineage mapping studies (36). Important distinctions between fetal and adult Leydig cells have been progressively mapped, revealing that synthesis of the androgen precursor, androstenedione, is different in fetal Leydig cells compared to adults. Whereas luteinizing hormone (LH) stimulates steroid biosynthesis in adult Leydig cells, fetal cells can be stimulated by corticotrophin-releasing hormone (CRH) and adrenocorticotrophic hormone (ACTH), and their responsiveness to LH is probably not attained prior to E17 in mice (37, 38). Single cell RNASeq datasets have also shed new light on where, and which, steroids and androgens are produced in the fetal testis, as discussed below.

### Sertoli Cells Are the Site of Testosterone Production in Fetal Mouse Testes

In the rodent testis, testosterone production occurs *via* the Δ4 steroidogenic pathway (**Figure 1B**, orange line) and concludes with conversion of androstenedione (A4) to testosterone (T) by the enzymes HSD17B3 (39) and HSD17B1 (40). The fetal and adult Leydig cell populations develop sequentially and are functionally distinct (41), so while T is produced in Leydig cells in adult mouse testes (42), fetal Leydig cells lack *Hsd17b1* and *Hsd17b3* and cannot convert A4 to T (39, 40). Instead, exclusive expression of *Hsd17b1* and *Hsd17b3* in fetal mouse Sertoli cells (43, 44) identifies Sertoli cells as the site of T synthesis in the fetal testis (33).

Since appropriate levels of steroids and steroidogenic enzymes during embryonic (E) development lay the foundation for the correct development of male sexual characteristics, factors that influence this process may illuminate important areas for research aimed at understanding the foundations of male reproductive disorders. Activin A (encoded by *Inhba*) is produced by fetal Leydig cells and is essential for normal embryonic mouse testis development. *Inhba* transcript levels in male testes increase directly after sex determination (E12.5) and the activin A dimer acts directly on receptors in Sertoli cells influencing their proliferation (30, 31, 45). Recently, our lab identified that activin A also positively regulates expression of *Hsd17b1* and *Hsd17b3* required to convert A4 to T; both are strongly reduced (E13.5 - E15.5) in the absence of activin A (*Inhba* KO) compared with wildtype controls (33). Analysis of intratesticular steroids demonstrated that a sustained decrease in *Hsd17b1* and *Hsd17b3* transcripts reduced conversion of A4 to T within E17.5 *Inhba* KO fetal testes; A4 levels were significantly increased and T tended to decrease. Additionally, the T/A4 ratio revealed a highly significant dose-dependent decrease in androgen production that correlated with reducing *Inhba* gene dosage (33). These data demonstrate that activin A fulfils a central role in determining local steroid levels and androgen production during fetal testis development. Lastly, Whiley et al. measured increased levels of the 11-oxygenated androgens, 11-KA4 (11-keto androstenedione, also named adrenosterone) and 11-KT (11-ketotestosterone) in *Inhba* KO testes (33). Since 11-KT can activate the androgen receptor (46), this finding could have important clinical significance.

### Steroidogenesis in the Human Male Fetus: What We Know and What Are It’s Vulnerabilities?

Production of the androgen T by the human fetal testis is essential for masculinization. Its release from the testis drives virilization of the Wolffian ducts to form the seminal vesicles and ejaculatory ducts, whereas the 5α-reduction of T to the more potent androgen dihydrotestosterone (DHT) is necessary for virilization of the male external genitalia (47). The first trimester human testes can produce low levels of T *via* the Δ4 pathway prior to a switch to the Δ5 pathway at the beginning of the second trimester (48). Thereafter, the human fetal testis produces high levels of T during the second trimester *via* the classical Δ5 steroidogenic pathway (**Figure 1B**, blue lines) (48, 49). This switch coincides with the maturation of fetal Leydig cells as they acquire their capacity to produce androgen precursors (48). The precursor androstenedione (A4)) is converted to T mainly by the 17β-hydroxysteroid dehydrogenase activity of HSD17B3 (50) (**Figure 1**) and while HSD17B1 can perform this conversion, it is less efficient compared to the mouse enzyme (51). In the human fetal testis, *HSD17B3* levels are comparable in first and second trimesters (48). However, the peak in T synthesis in the second trimester appears due to fetal Leydig cells becoming fully differentiated and acquiring the ability to synthesize androgen precursors (48).

Leydig cells are the major steroidogenic cells of the human fetal testis, expressing a wide range of steroid biosynthetic enzymes, and their differentiation coincides with elevated testicular T levels (49). Thus, these cells are often assumed to be responsible for androgen biosynthesis. However, as described in the section “Sertoli Cells Are the Site of Testosterone Production in Fetal Mouse Testes”, although the precursor steroids and A4 are produced by fetal Leydig cells in rodents, the conversion of A4 to T takes place in the Sertoli cells (**Figure 1**). Whether this also occurs in humans is not established (48). To address this possibility, we interrogated published single cell RNASeq datasets to determine in which cell types key steroidogenic enzymes are synthesized in mouse and human testes (35, 44). The expression patterns for *HSD17B3* and *HSD17B1* in human testicular cells provide strong evidence that, as in mice, human Sertoli cells are likely to be the primary site of androgen (T) synthesis in the second trimester fetal testis (**Table 1**; **Supplementary Figure 1**). Interestingly, human scRNASeq data suggest that only a small subset of human fetal Leydig cells produce androgen precursors between weeks 14–18 of gestation [**Supplementary Figure 1** (35)]. This speculation is aligned with evidence that human fetal interstitial and Leydig-like cells are a heterogenous population (49).

**Table 1:** mRNA expression patterns of key steroidogenic enzymes in fetal mouse and human testis cells^a^.

Gene symbol (mouse, human)	Mouse fetal testis cell expression^b^	Human fetal testis cell expression^c^
*Cyp11a1, CYP11A1*	Leydig cells	Leydig cells > Sertoli cells
*Hsd3b1, HSD3B1*	Leydig cells	Not available
*Cyp17a1, CYP17A1*	Leydig cells	Leydig cells
*Hsd17b3, HSD17B3*	Sertoli cells	Sertoli cells
*Hsd17b1, HSD17B1*	Sertoli cells	Leydig and Sertoli cells
*Cyp11b1, CYP11B1*	Leydig cells	Not detected
*Hsd11b2, HSD11B2*	Stroma, peritubular myoid	Leydig cells
*Akr1c6, AKR1C3*	Not present	Not detected

a Predominant site of expression as determined by UMAP plots from the relevant dataset (see **Supplementary Figure 1**)

b Data from E18.5 mouse testis cells according to Tan et al. (44)

c Data from human testis cells at 12, 15 and 16 weeks post-fertilization (14, 17 and 18 weeks gestation) according to Guo et al. (35)

Recent studies have revealed that alternative androgen production by the so-called “backdoor pathway” is also important for virilization of the human male fetus (52, 53). This pathway begins with progesterone, probably originating largely from the maternal side of the placenta (**Figure 1A**), followed by sequential biosynthetic steps in the adrenal glands and liver, that ultimately produce androsterone (53). This androgen is present in the human fetal male circulation at levels similar to testosterone (53). Thus, masculinization of the human male fetus depends on androgens produced *via* the classic and backdoor pathways, both within and outside of the testis. It involves a complex interplay of steroidogenic enzymes expressed in multiple tissues to produce testosterone, DHT and androsterone that act within different organs to control human fetal urogenital tract development (49, 52, 53).

Finally, as noted above, certain 11-keto steroids have the capacity to contribute to androgen action in the human fetal testis and other androgen-responsive tissues. Originally identified as androgens in fish, the 11-keto androgens 11-keto testosterone (11-KT) and 11-keto DHT (11-KDHT) are potent agonists of the human androgen receptor (46, 54, 55). They are likely to be synthesized in human peripheral tissues from the abundant adrenal steroid 11b-hydroxyandrostenedione (11OHA4) (**Figure 1B**) (46, 54). CYP11B1 is a key enzyme in 11-keto steroid biosynthesis (**Figure 1B**) and is expressed in mouse and human testis (**Table 1**; **Supplementary Figure 1**). This enzyme can be stimulated by hCG in immature mouse Leydig cells leading to an increase in testicular levels of 11-KT (55). In mice, 11-KT is shows similar androgen activity to T, suggesting it is an important bioactive androgen (55). The enzyme responsible for the biosynthesis of 11-keto androstenedione (11-KA4), HSD11B2 (**Figure 1B**), is expressed in both mouse and human testis cells (**Table 1**; **Supplementary Figure 1**) whereas the enzyme that converts 11-KA4 to 11-KT (AKR1C6 in mice and AKR1C3 in humans (**Figure 1B**), is not expressed in the testis (**Table 1**, **Supplementary Figure 1**). These data suggest that fetal mouse and human testes express the enzymes that produce 11-KA4 and that this precursor can be converted to the androgen 11-KT in peripheral tissues. We have detected both 11-KA4 and 11-KT in fetal mouse testes, indicating that 11-KT could act as bioactive androgen during fetal testis development (33). Importantly, we showed the levels of these keto steroids are responsive to activin A deficiency; reduced conversion of A4 to T led to increased levels of A4 that were accompanied by increased testicular levels of 11-KA4 and 11-KT (33). Thus, in activin A-deficient mice, the reduced capacity to produce the androgen T, as a consequence of reduced *Hsd17b1* and *Hsd17b3* expression in Sertoli cells, is accompanied by an increased capacity to produce the androgen 11-KT (33). Further studies are needed to understand the contribution of 11-KT to androgen action in the human fetal testis and in the masculinization of the male fetus.

## Conclusions: Why Does This Matter?

The complexity of human fetal steroidogenesis means that there are multiple cell types and thus sites vulnerable to disruption that could alter androgen levels and activities in different fetal tissues. In the mouse testis, activin A regulation of the T biosynthetic enzymes *Hsd17b1* and *Hsd17b3* in Sertoli cells is now known to alter the ratio of A4 to T and the production of keto-steroids with androgenic bioactivity (**Figure 1B**), and this is likely to be important to some extent in humans (**Table 1**). Changes to activin A bioactivity at the start of gonad development, as in the condition of pre-eclampsia, would be ultimately expected to influence adult fertility and development of other organs; this may also affect the developing ovary. The fact that fetal T is produced by the Sertoli cells and thus T levels are not an accurate measure of fetal Leydig cell function needs to be more widely appreciated. Recent data showing that androgens in the human male fetus testis can originate *via* different biosynthetic pathways adds complexity to the mechanisms *via* which external and intrinsic factors could modulate androgen action during male fetal development. This knowledge will help to identify a roadmap of developmental vulnerability to environmental exposures, focussed appropriately on the key affected cell types. This should ultimately be used to develop strategies that can protect reproductive health.

## Author Contributions

KL designed content, co-wrote text, and edited text and figure. PW co-wrote and edited text, designed and produced figure. LO’D co-wrote text, and edited text and figure. All authors contributed to the article and approved the submitted version.

## Funding

This work was supported by funding from the NHMRC (Ideas 1181516).

## Conflict of Interest

The authors declare that the research was conducted in the absence of any commercial or financial relationships that could be construed as a potential conflict of interest.

## Publisher’s Note

All claims expressed in this article are solely those of the authors and do not necessarily represent those of their affiliated organizations, or those of the publisher, the editors and the reviewers. Any product that may be evaluated in this article, or claim that may be made by its manufacturer, is not guaranteed or endorsed by the publisher.
