# Supplementary material for: Activin A and Sertoli Cells: Key to Fetal Testis Steroidogenesis
Source: Front Endocrinol (Lausanne). 2022 May 24;13:898876. doi: 10.3389/fendo.2022.898876 (PMC9171382; doi:10.3389/fendo.2022.898876)

Supplementary Figure 1

Single cell RNASeq datasets from mouse (1) and human (2) fetal testicular cells were investigated and explored using the Seurat (3) package (https://satijalab.org/seurat/, R package v4.0.4). **1A.** Data from mouse testis cells at E18.5 days was retrieved from the Gene Expression Omnibus (GEO) repository (Accession GSE130593). The left-hand panel indicates the clustering of mouse testicular cells into specific cell lineages based on marker gene expression (1) and the right-hand panel indicates the mRNA expression patterns of key steroidogenic enzymes as indicated in Table 1. **1B.** Data from human testis cells at 12, 15 and 16 weeks post-fertilization (14, 17 and 18 weeks gestation) was retrieved from the Gene Expression Omnibus (GEO) repository (Accession GSE143356). The left-hand panel indicates the clustering of human testicular cells into specific cell lineages based on marker gene expression (2) and the right-hand panel indicates the mRNA expression patterns of key steroidogenic enzymes as indicated in Table 1.

References:

1. Tan K, Song HW, Wilkinson MF. Single-cell RNAseq analysis of testicular germ and somatic cell development during the perinatal period. Development. 2020;147(3)
2. Guo J, Sosa E, Chitiashvili T, Nie X, Rojas EJ, Oliver E, et al. Single-cell analysis of the developing human testis reveals somatic niche cell specification and fetal germline stem cell establishment. Cell Stem Cell. 2021;28(4):764-78 e4

3. Hao Y, Hao S, Andersen-Nissen E, Mauck WM, 3rd, Zheng S, Butler A, et al. Integrated analysis of multimodal single-cell data. Cell. 2021;184(13):3573-87 e29.

Supplementary Figure 1A.

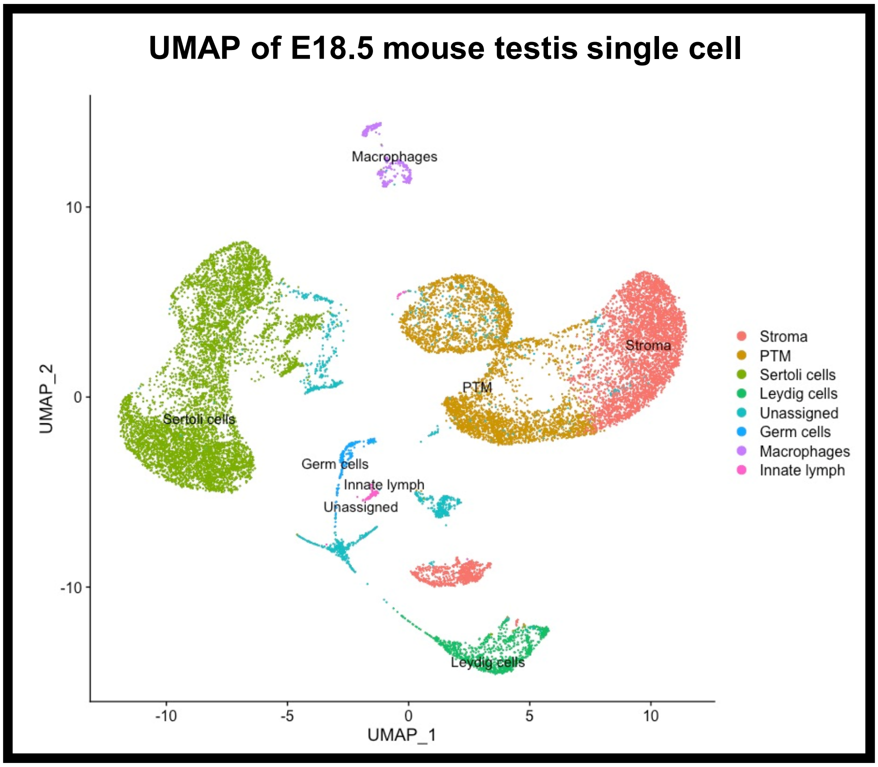

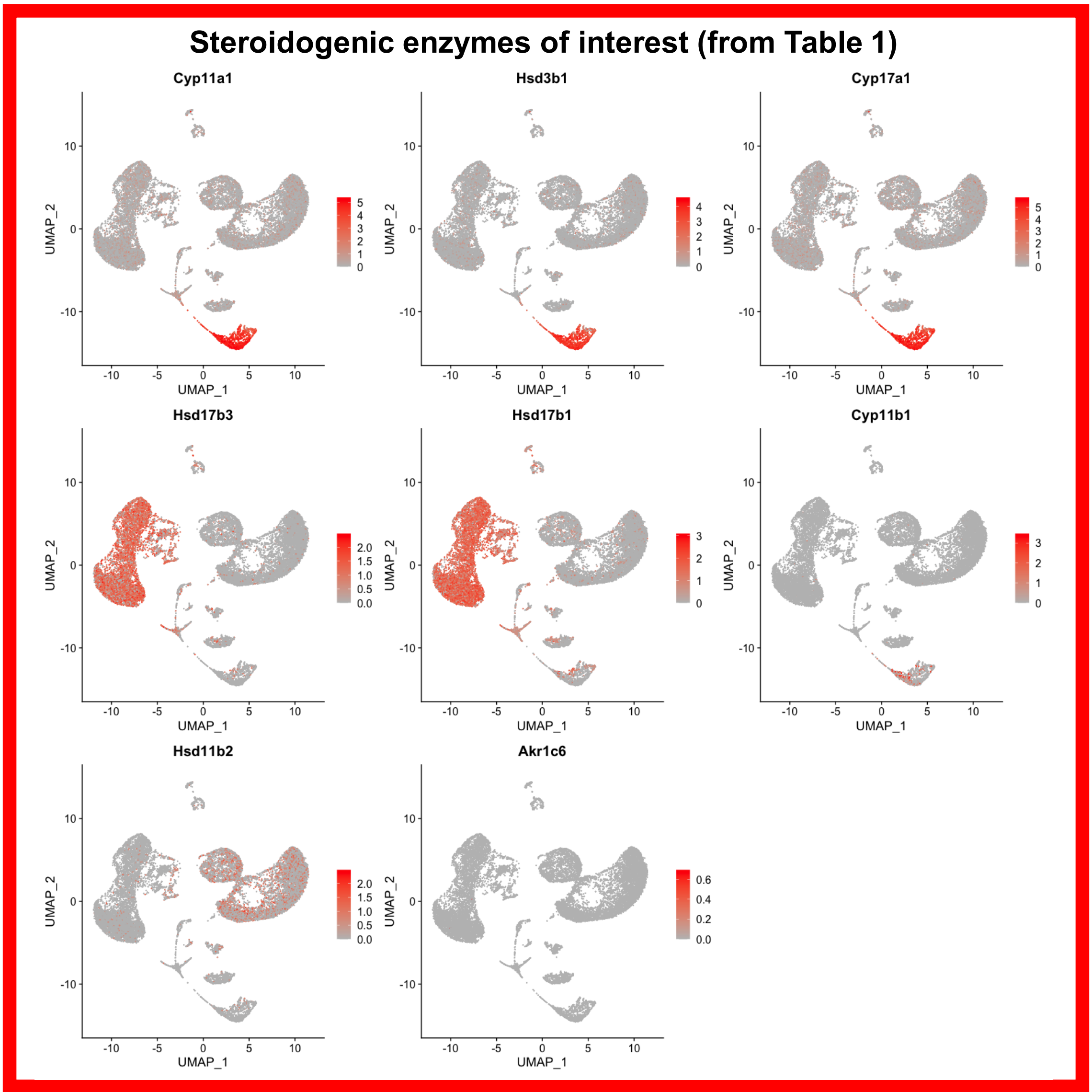

Supplementary Figure 1B.

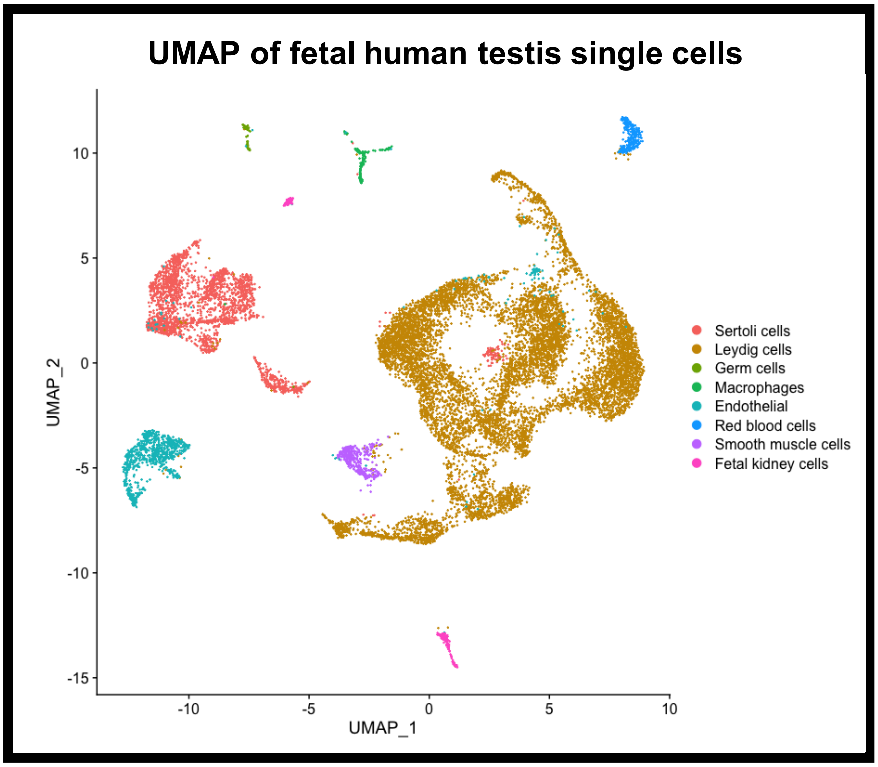

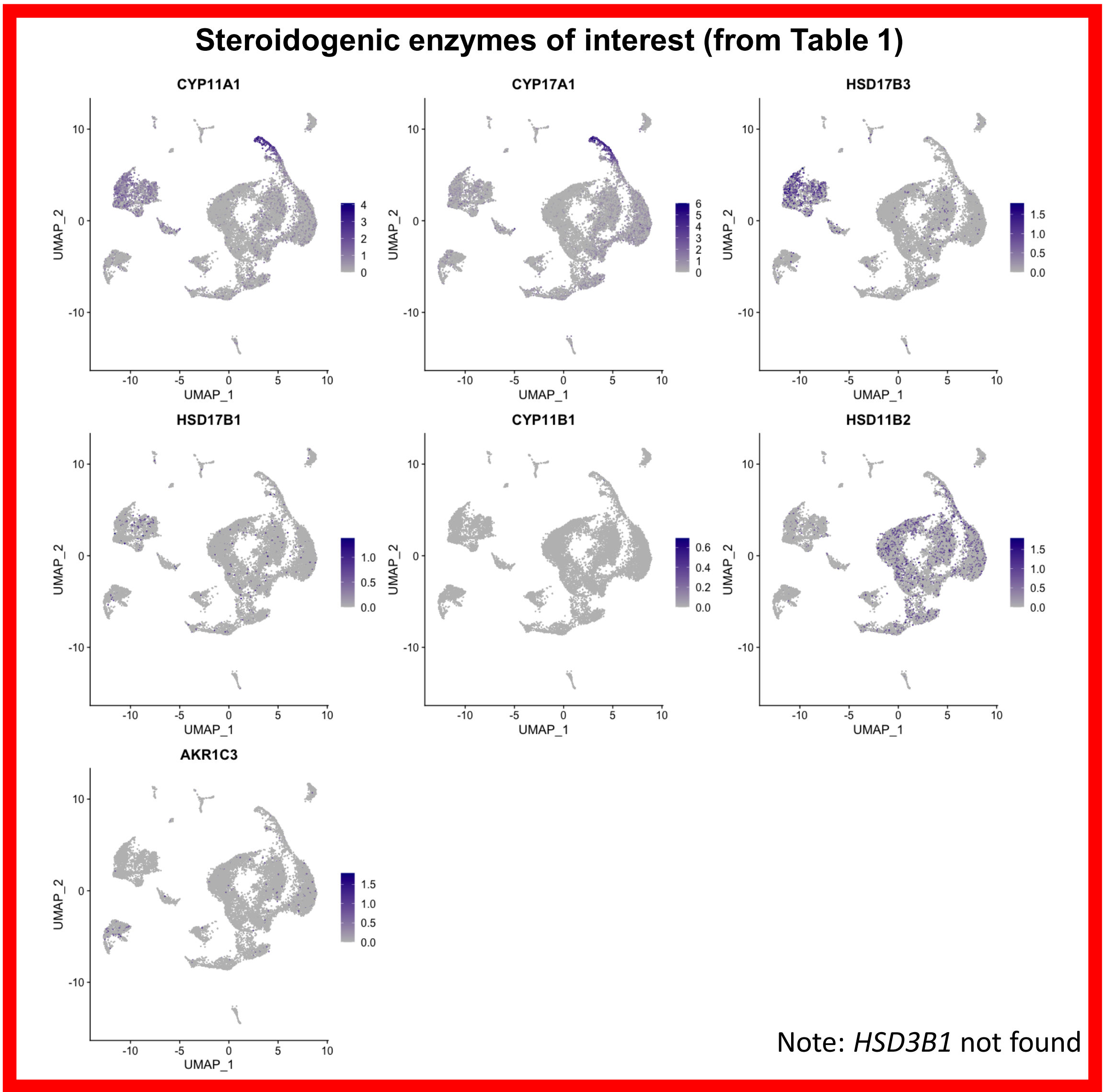

Supplement: Supplementary file 1 [file DataSheet_1.pdf]
